# Special Issue “Advances in Drug Discovery and Synthesis”

**DOI:** 10.3390/ijms26020584

**Published:** 2025-01-11

**Authors:** Lidia Ciccone, Susanna Nencetti

**Affiliations:** Department of Pharmacy, University of Pisa, Via Bonanno 6, 56126 Pisa, Italy; susanna.nencetti@unipi.it

In modern medicinal chemistry, drug discovery is a long, difficult, highly expensive and highly risky process for the identification of new drug compounds [1,2,3,4].

The word drug refers to chemicals administered to humans or animals, with a medicinal scope. Medicinal chemistry is a discipline focused on the synthesis and analysis of drugs (pharmaceutical chemistry), but it also investigates the interactions between the drugs and their biological targets [5,6,7]. The drug discovery process is characterized by different steps starting with target identification, and moving through the hit to lead, lead optimization, and candidate selection steps to pre-clinical and clinical trials [8], as shown in Figure 1.

Drug discovery is a process in which many disciplines are involved, such as organic chemistry, biochemistry, pharmacology, genetics, immunology, toxicology, molecular biology, X-ray crystallography, and computational chemistry [9,10,11]. Drugs can be of natural or synthetic origin and elicit their effects through interactions with specific targets in the human body. Drug discovery can be carried out using different approaches. The oldest one is by serendipity, as can be seen in the discovery of penicillin, chlordiazepoxide, and cyclosporin. Another approach is the chemical modification of known drugs or natural products to improve their affinity and selectivity, reduce their toxicity and side effects, and improve their stability and water and lipid solubility [12,13]. In recent years, the repurposing of existing drugs has emerged as a successful alternative approach to drug discovery, in which drugs target diseases different from those determined in their original clinical indication [5,14,15,16]. This approach has the advantage of reducing the cost and time of drug discovery [17]. Rational drug design is a smart and cheap strategy that uses the information derived from X-ray crystal structure and molecular modeling [18,19,20,21,22].

Recently, artificial intelligence (AI) has accelerated all aspects of the drug discovery process, predicting the 3D structure of proteins, their interaction with drugs, and the activity and synthesis of new molecules [23,24,25].

Considering the long path of the drug discovery process, a new molecule, before becoming a drug, can be studied for decades. Moreover, the drug candidate may fail during pre-clinical or clinical trials. Over the years, drug discovery has developed advanced methodologies, combining experimental results with computational studies in order to guide the discovery and optimization of drugs [26,27].

In this Special Issue “Advances in Drug Discovery and Synthesis”, we collected nine scientific works that contribute to the drug discovery process to find treatments for several diseases, such as cancers, diabetes, infections, and neurodegeneration.

Despite the great results obtained in the treatment of various cancers, in which new therapies can significantly improve patients’ quality of life and the patient survival rates, glioblastoma multiforme (GBM), the most aggressive primary brain tumor, lacks an effective treatment [28,29,30].

In this context, Gallego-Yerga et al. suggested tubulin, a proven target protein in the field of anticancer drug discovery, as a new potential target for the treatment of GBM [31]. Among the different binding sites reported for tubulin, the authors focused their attention on the development of ligands targeting the colchicine binding site. To achieve their aim, they developed a computational protocol, and the results drove the design and synthesis of a new tetrazole-based series of tubulin inhibitors as potential anticancer compounds. In vitro studies showed that the new derivatives were safe to non-tumor cells and exhibited good anticancer activity in the GBM cell model. Regarding the mechanism of action of the studied compounds, the experimental evidence suggested that they were able to interact with tubulin-blocking cells at the G2/M phase, inducing apoptosis [31].

Often, anticancer drugs are characterized by their low solubility in water solution, and this aspect decreases their bioavailability, reflecting reduced in vivo activity [32,33,34]. Thus, a possible solution to increase the solubility and bioavailability of promising anticancer drugs could be to incorporate them into cyclodextrin systems [35]. In their research, Adamus-Grabicka A.A. et al. identified several potential anticancer compounds structurally composed of chromanone/flavanone groups and nitrogen atoms (spiro-1-pyrazolines). A barrier to the study of these derivatives as potential anticancer drugs is their low solubility in water. The research group selected some promising derivatives and synthesized inclusion complexes with specific cyclodextrins with high water solubility [36]. The compounds showed a very promising profile in breast and endometrial cancer cells and three of them were investigated in more depth. The authors suggested that their pro-apoptotic properties could be related to their ability to generate radical oxygen species (ROS) in cancer cells [36].

In the field of advanced drug discovery, it is natural to think about multi-target compounds [37,38]. The multi-target approach was proposed almost thirty years ago when a group of researchers proved that it would be possible to design a compound endowed with a multi-target mechanism of action [39,40,41]. Over the years, this strategy has been applied to several diseases, such as inflammation, cancer and, in particular, Alzheimer’s diseases (AD) [42,43,44,45]. AD is a multifactorial neurodegenerative disorder that is still lacking an effective cure [46,47]. In this context, Drozdwska D. et al. reported the synthesis and characterization of a new series of benzamides derivatives as multi-target compounds that can be used as potential inhibitors of acetylcholinesterase (AChE) and β-secretase (BACE1) [48]. Among the synthesized compounds, derivative N,N′-(1,4-phenylene)bis(3-methoxybenzamide) was identified as the best one because it was able to inhibit both AChE and BAC1 in the micromolar range and a molecular docking study suggested it contained a possible binding mode with the two enzymes [48].

As was previously mentioned, bioactive molecules derived from nature have played a fundamental role in the drug discovery process and, through the years, natural products have gained more and more attention [49,50,51,52]. One of the limits related to the use of natural compounds in therapy is their low bioavailability, which suggests reduced pharmacokinetic properties [53,54,55]. Thus, scientists often propose chemical structural modifications of natural bioactive molecules to improve their solubility and stability. Recently, Álvarez-Almazán S. et al. reported the synthesis of two semisynthetic glycyrrhetinic acid (GA, pentacyclic triterpene extracted from the plant Glycyrrhiza glabra) derivatives as potential anti-diabetic molecules to act against Protein Tyrosine Phosphatase 1B (PTP1B) and α-glucosidase enzymes [56]. In vitro, in silico, and in vivo studies suggested that both semisynthetic compounds are safe and one of them showed a promising profile, suggesting the need for further investigation [56]. In this Special Issue, we present another example of synthetic molecules derived from a natural compound: tabamide A (TA0) isolate from Nicotina tabacum. Yong Shin S. et al. synthesized TA0 and derivatives and investigated their activity against influenza virus. The study identified some functional groups that are fundamental for antiviral activity. Among the synthetized TA0 analogs, one compound showed a promising profile in an in vivo mouse model [57].

In modern medicinal chemistry, a relevant role in the drug discovery process is played by computational methods, which can reduce the time and cost required in the discovery and optimization of ligands [58,59,60]. The in silico prediction of new small ligands directed towards a specific target, as well as an virtual investigation of their biological profile, is considered a valid approach to reduce failures in drug discovery [61,62]. In the following paragraph, we present four works collected in our Special Issue in which computational methods guide the research study. Milusheva M. et al., using in silico prediction results, synthesized a series of promising antispasmodics compounds. These theoretical studies were followed and consolidated by in vitro tests suggesting that the new anthranilic acid hybrid is an excellent candidate for future antispasmodic drug development [63]. Kolić D. and Šinko G. evaluated the inhibitory activity of mefentrifluconazole and pyraclostrobin on human acetylcholinesterase (AChE) and human butyrylcholinesterase (BChE). Then, to understand their dual effect, in silico studies were conducted on both enzymes. The results suggested that mefentrifluconazole and pyraclostrobin could represent a threat for human cytochrome, but also for AChE, by disrupting cholinergic neurotransmission, as well as presenting a threat to the related BChE [64].

The drug discovery process also has a relevant role in agriculture because active chemical compounds are important in preserving plant health. In this Special Issue, we present the work of Koyama T. et al., who studied small molecules that are able to control the ethylene response in *Arabidopsis*. They identified a derivative using the thiourea scaffold that represents a promising compound for further development as a potent crop-controlling chemical [65].

Finally, we would like to shed light on microRNAs (miRNAs). Recently, miRNAs have gained great attention as a potential novel class of drugs because of their link to several human diseases [66,67,68]. In recent decades, studies have reported that miRNAs could also be useful as diagnostic biomarkers or therapeutic targets for neurodegenerative diseases such as glaucoma. Zhang R. et al. conducted a bibliometric analysis in the field of microRNAs in glaucoma research, with the aim of presenting the current trends and research hotspots and presenting the emerging trends in this field [69].

## Figures and Tables

**Figure 1:**
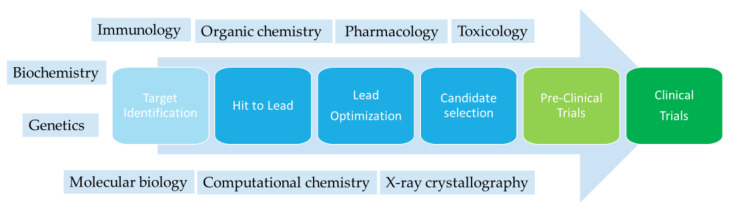
Scheme of the drug discovery process and the disciplines involved.

## Data Availability

Data Availability Statements are available in the Section “MDPI Research Data Policies” at https://www.mdpi.com/ethics.
